# Design of the DAVOS Study: Diabetes Smartphone App, a Fully Automatic Transmission of Data From the Blood Glucose Meter and Insulin Pens Using Wireless Technology to Enhance Diabetes Self-Management—A Study Protocol for a Randomized Controlled Trial

**DOI:** 10.1177/19322968221075333

**Published:** 2022-04-08

**Authors:** Franziska Grosser, Sandra Herrmann, Maxi Bretschneider, Patrick Timpel, Janko Schildt, Markus Bentrup, Peter E. H. Schwarz

**Affiliations:** 1 Department of Prevention and Care of Diabetes, Department of Medicine III, Technische Universität Dresden, Dresden, Germany; 2 Emperra GmbH E-Health Technologies, Potsdam, Germany

**Keywords:** diabetes data management, diabetes mellitus, HbA1c, randomized controlled trial, self-management, digital health

## Abstract

**Background:**

In the treatment of diabetes mellitus, the challenge is to integrate adequate self-management into clinical care. Customization including goal setting, monitoring, and feedback could be achieved through digitization. Digital linking between different devices could simplify and promote self-management. The aim of this study is to evaluate the outcome of diabetes treatment assisted by a digital health application compared with standard diabetes therapy.

**Methods:**

The DAVOS study is a 6-month-period prospective, multicentric, randomized controlled trial. In total, 154 diabetes patients (age ≥18; treated with insulin) will be recruited and randomized into control group or intervention group. Both groups will receive standard diabetes care. The intervention group will additionally use a diabetes app. HbA1c value will be monitored on three separate defined visits. Primary endpoint is the overall reduction of HbA1c value. Secondary endpoints (eg, usability of the app) will be determined through patient-reported outcome questionnaires.

**Discussion:**

Through enhanced interaction of health care professionals, providers of the app, and patients, the study aims to demonstrate improvement in the self-management of diabetes. As part of the closure management, all patients will be invited to use the examined application after completion of the study. The DAVOS study will be conducted in accordance with the valid version of the present study protocol and the internationally recognized International Conference on Harmonization–Good Clinical Practice (ICH-GCP) Guidelines. Special attention will be paid to European, national, and regional requirements for the approval, provision, and use of medical devices. The study was registered in the German Register of Clinical Trials (DRKS) with number DRKS00025996.

## Introduction

Diabetes mellitus has increasingly become a widespread disease.^1^ As diabetes is a chronic disease, the term “cure” as a goal may not be entirely accurate.^2^ As part of nonpharmacological interventions, individual self-management with constant professional supervision is a necessity.^3^

Digital health applications (DHAs) have the potential to improve access to relevant target groups, provide individualized feedback, and overall improve quality of care.^4^ However, patients cannot simply be expected to comply but should be provided with products that promote compliance through ease of use and motivation for incorporating self-management into daily life.^5^

The evidence on the effectiveness of DHAs for monitoring and self-management support in patients with diabetes can be considered a weak and inconclusive.^6,7^ The inability to connect devices from different manufacturers (eg, pen, glucometer, and app) creates an evolving challenge for providers. This compromises the ability to appropriately combine effective treatment options and complicates patients’ self-management.^8^

Despite these challenges for developers, health care professionals (HCPs), and patients,^9^ DHAs hold a great potential to improve diabetes self-management, largely due to improved monitoring, immediate and ongoing feedback, and motivation.^7,10-13^

The aim of this study is to assess the effectiveness of a DHA on clinical outcomes in patients with diabetes under real-life conditions. Collected data on blood values, empowerment, self-management, mental health, and quality of life will provide the necessary basis for certification of the investigated smartphone app as an official DHA in Germany following the “DiGA Fast Track evaluation” as a guideline for the approval.^14^

## Methods

### Trial Design

**Figure 1.**
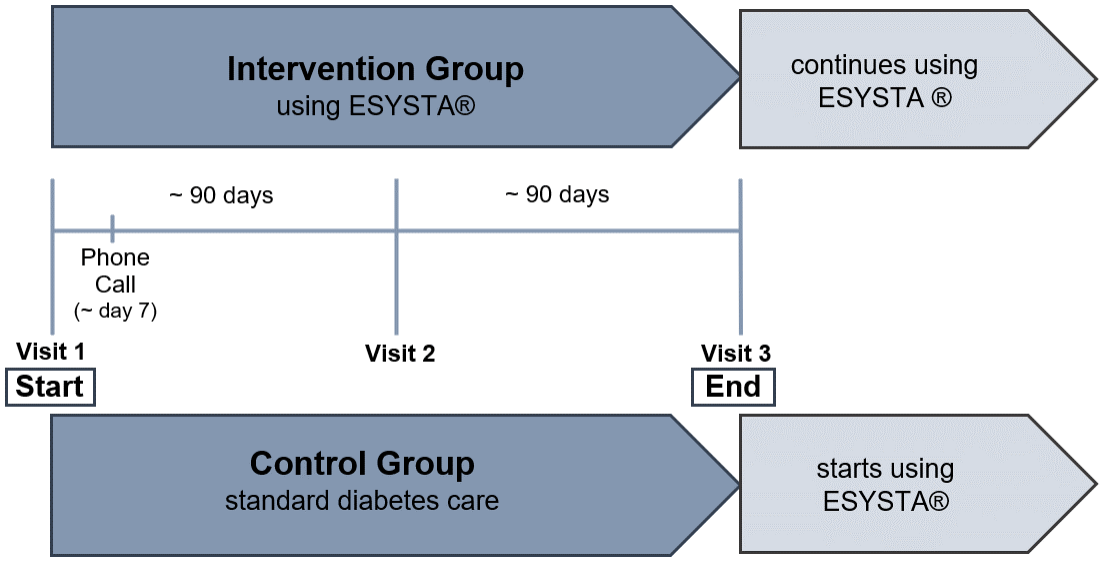
Schematic design of the DAVOS trial (E**V**aluation **O**f the Impact of E**S**ysta on Hba1c in Patients with Type 1 and Type 2 Diabetes mellitus in **DA**ily practice).

A prospective, multicentric, randomized controlled trial (RCT) will be conducted over a period of 6 months to investigate the effect of a DHA on HbA1c levels in patients with type 1 and type 2 diabetes mellitus. To this end, we will compare two groups of patients fulfilling the inclusion criteria (see Table 1): The control group (CG) will only receive the standard diabetes care and the intervention group (IG) will additionally use the DHA to monitor diabetes-relevant values using various graphs and observe potential changes in these endpoints. Each group will consist of at least 77 patients and will be supervised for a minimum of 180 days. The schematic design of the DAVOS trial (E**V**aluation **O**f the Impact of E**S**ysta on Hba1c in Patients with Type 1 and Type 2 Diabetes mellitus in **DA**ily practice) is shown in Figure 1.

**Table 1.** Inclusion and Exclusion Criteria in the DAVOS Trial.

*Inclusion criteria*
• Diabetes mellitus type 1 or 2 • ≥18 years old • Treated with insulin • HbA1c at baseline ≥7.5% and ≤11.0% • Not having used the investigated DHA in the past 12 months • Willing to use a smart glucometer and/or smart insulin pen • Internet access, smartphone/computer compatible with the DHA • Digital literacy to use a smartphone adequately
*Exclusion criteria*
• Using other apps linked to a BG meter or using an insulin pump or continuous glucose monitoring • Having used the investigated DHA in the past 12 months • <18 years old • Impairments (also mental impairments, eg, psychotic disorders) • Patients with home nursing (assisting BG testing) • Current participation in a weight loss program • Current participation in another study • Steroid therapy within the past three months (no exclusion criterion if used topically or inhaled less than five times a week) • Blood pressure ≥200 mm Hg at screening • BMI ≥40 kg/m² • Anemia (according to the WHO definition^15^) • GFR ≤40 mL/min • Current or planned pregnancy, breastfeeding women • Alcohol or drug abuse (within the past three months) • Employees of Emperra GmbH E-Health Technologies (or other institutions being involved in the trial)

Abbreviations: BG, blood glucose; BMI, body mass index; DHA, digital health application; GFR, glomerular filtration rate; WHO, World Health Organization.

**Figure 2.**
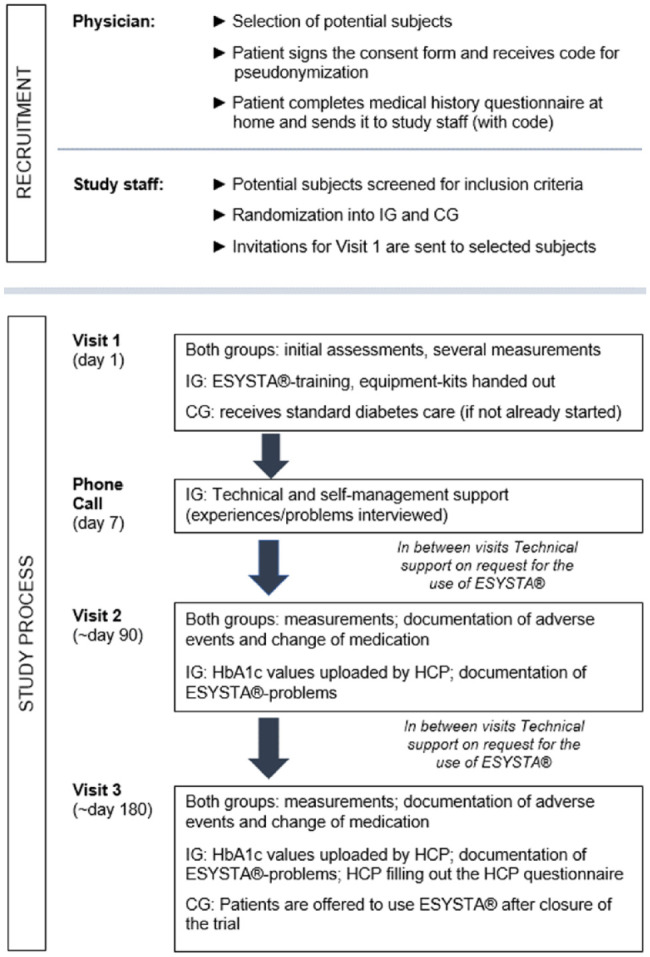
Visit schedule. Abbreviations: CG, control group; HCP, health care professional; IG, intervention group.

The decline of HbA1c value is the primary endpoint of the study. The values will be monitored on three visits: in the beginning, after 90 days, and after 180 days. Furthermore, the IG will receive an additional telephone call seven days after the first visit to resolve any technical barriers, including problems with connected hardware (which participants will receive after Visit 1). All visits are specified in Figure 2.

The study as a prospective, multicentric, randomized, controlled trial was approved by the German Federal Institute for Drugs and Medical Devices (BfArM) on July 5, 2021, and will be submitted to the Independent Ethics Committee.

Five different study sites, guided by physicians with experience in digital diabetes management (according to Good Clinical Practice [GCP]), are planned for the execution of the study. Both physicians and practice staff are briefed to collect the necessary values for the study. Patients will visit the office and be treated by their physician as usual, except that they will be attending the trial visits in addition.

### Participants

#### Eligibility criteria

Included in the trial are insulin-treated diabetes patients, who have agreed to the conditions of the study. All patients should be willing to improve self-management. Main prerequisite for participation is diabetes mellitus (type 1 or 2) treated with insulin. Previous duration of diabetes and insulin treatment are irrelevant. Patients, who do not use insulin yet but—according to HCP—can profit from insulin therapy, can also be included in the trial. Diabetes control is not important for inclusion as long as the HbA1c value is ≥7.5% and ≤11.0%.

For all inclusion/exclusion criteria, see Table 1.

#### Recruitment and randomization

Recruitment of the patients will take place in routine clinical practice in several medical practices/study centers in Germany.

A simple randomization will be performed with the generation of a randomization list.^16^ For this purpose, participating physicians will perform individual randomization by screening potential participants for their inclusion criteria by study personnel. After signing the informed consent form and the anamnesis questionnaire, patients then will be randomized using the predesigned randomization list.

Patients who meet all inclusion criteria will then receive an invitation to the first visit by post. Later, participants of the IG will be asked to sign another consent form to use the DHA.

As this is not a blinded study, the results of randomization cannot be hidden from the study centers. The division into IG and CG will be obvious to staff and participants.

Pseudonymization will be performed using a code that patients will receive upon consenting to the study. The data will not be passed on to third parties.

### Intervention

Both groups are offered standard diabetes therapy, as they should not experience any deficiency in professional care. All participants are encouraged to measure blood glucose (BG) ideally before meals, two hours after meals, and before going to bed, but at least three times a day during the entire study. The customer service for telephone support will be available at any time.

#### Control group

An exclusion of the CG from using the DHA is necessary, but it is not possible to monitor the use of other digital tools for diabetes self-management. Therefore, it will be advised not to use any assisting digital programs during the study. By finishing the trial, every patient will be invited and guided to use the DHA.

#### Intervention group

The IG will apply a DHA, named ESYSTA®. The patients will be taught to use both components, app and portal, which automatically synchronize with each other. The DHA system is supposed to replace a paper-based diabetes diary.

By using a compatible blood glucose meter (BGM) and/or insulin pen, patients can import BG or insulin data by connecting with the app via Bluetooth®. The system enables the connection of BGMs that are compatible with the “Glucose Profile” standard in Version 1.0 of the “Bluetooth® Special Interest Group.” This standard uses “Bluetooth® Low Energy” as transmission technology. A list of compatible devices is provided to the participants.^17^ It is also possible to document values manually and even when the app is offline.

**Figure 3.**
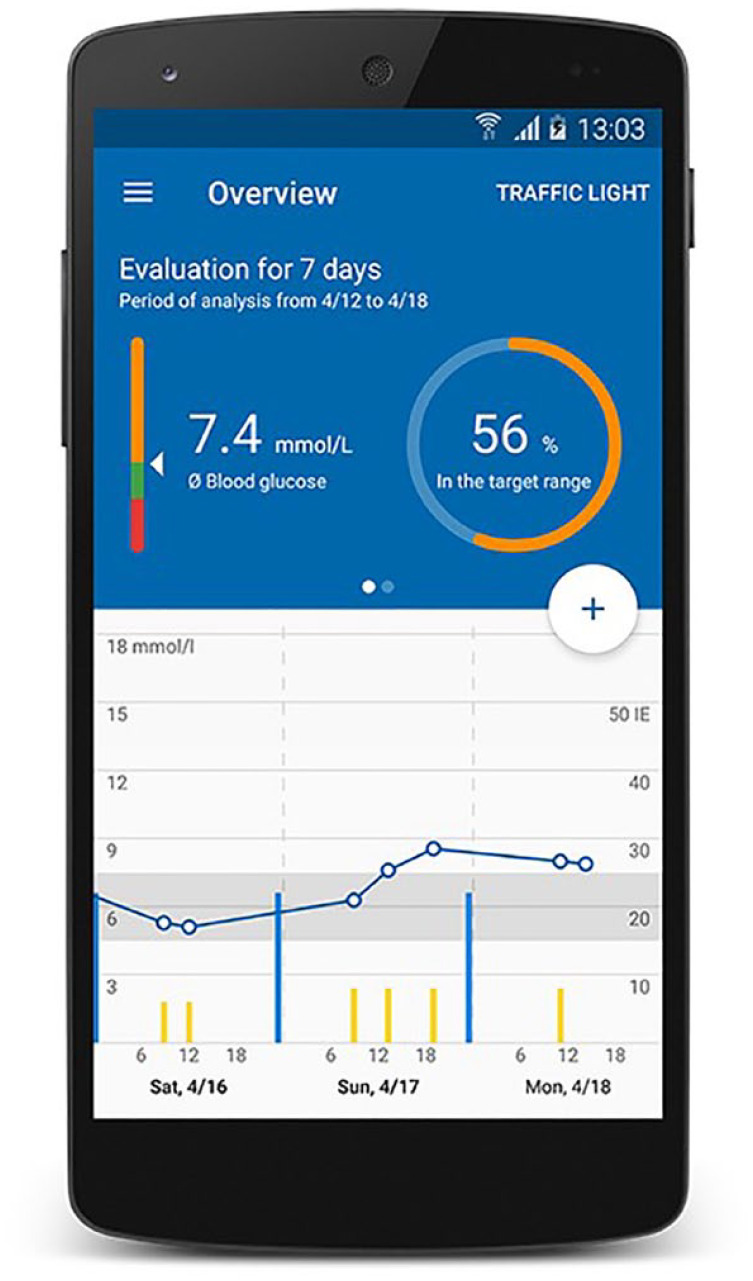
User interface of the app.^18^

The app offers a detailed daily display as well as a daily BG history (3-day or 7-day view) so patients can review their daily routine for themselves. A target range analysis and a traffic light provide quick information about the metabolic setting to increase motivation (Figure 3).

**Figure 4.**
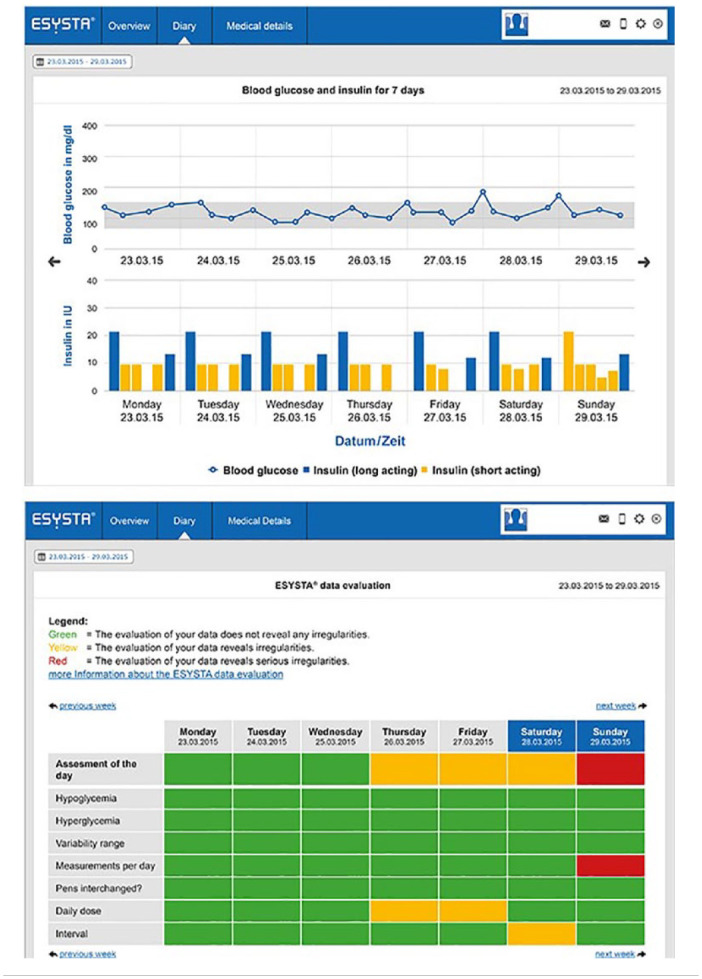
User interface of the portal.^19^

In the portal, more detailed graphics and tables than in the app are available (Figure 4). The traffic light system is also used here, for example, in a table to monitor hypoglycemia or hyperglycemia, measurements per day, and other points of interest. In addition, critical values and incorrect dosing can be quickly detected with graphics for insulin and BG. Both patient and doctor have access to the portal. Moreover, HCPs can import data from the portal into their practice or hospital IT system to avoid data loss or transmission errors. A protected exchange of messages between patient and physician through an integrated e-mail system is also possible via the portal (in addition to routine appointments and telephone consultations). Health care professionals are instructed to use the portal during the study as part of the intervention.

Patients in the IG will be provided a BGM compatible with the DHA, which they may keep after closure of the trial. Those patients who already own a compatible device have the option to use their current BGM for the study. If needed, the participants will also receive a kit with standard lancing device, single-use lancets, box of test strips, and compatible insulin pens.

### Measures and Questionnaires

Primary endpoint of the study is the change of HbA1c during the use of the DHA. The main hypotheses are listed in Table 2. Measurement of the HbA1c value, blood pressure, waist circumference as well as weighing and pregnancy test will take place in every visit in both groups. Blood samples will be tested in the local laboratory of each study site.

**Table 2.** Hypotheses of the Clinical Trial.

Hypothesis	Indicator	Relation
H0	Any	Is having no effect
H1	HbA1c	Is superior and has a clinical meaningful^a^ impact on lowering the HbA1c compared with standard diabetes care
H2	Hypoglycemic events	Is superior and has an impact on lowering hypoglycemic events compared with standard diabetes care
H3	Self-management	Is superior and improving self-management of diabetes patients compared with standard diabetes care
H4	Mental health	Is superior and reduces symptoms that represent a risk potential for secondary diseases such as depression compared with standard diabetes care

a HbA1c difference of 0.3 HbA1c % points.^20,21^

There will be several patient-reported outcome (PRO)^22-25^ questionnaires for evaluating changes in empowerment, self-management, mental health, quality of life, and other outcomes. The first questionnaire on personal data, medical history, and inclusion criteria as well as all further questionnaires will be completed via paper pencil.

All endpoints with matching questionnaires are listed and explained in Table 3.

**Table 3.** Predefined Endpoints.

Hypothesis	Primary endpoint	Data collection/questionnaire
H1	Change of HbA1c during the use of DHA	Data from HbA1c testing at the visit
Hypotheses	Secondary endpoints	Data collection/questionnaires
H1	Proportion of patients reaching their HbA1c treatment goals <7.0% during the use of DHA	Data from HbA1c testing at the visit
	Proportion of patients reaching their HbA1c treatment goals <6.5% during the use of DHA	
	Achieving a higher proportion of patients reaching the HbA1c treatment goals compared with CG	
H2	Frequency of severe and nonsevere hypoglycemic episodes (ADA recommendations: <70 mg/dL risk of hypoglycemic episodes, <54 mg/dL clinically important hypoglycemic episodes^26^)	Data from patient’s recollections (using DHA or Diary)
	Frequency of severe hyperglycemic events (ketoacidosis/nonketotic hyperosmolar coma)	Data from patient’s recollections (using DHA or Diary)
H3 H4	Changes in motivation and patient self-management	Measured by daily conducted BG values (DHA or Diary) and questionnaires:SDSCA^27^ collects data on self-management (including diet, exercise, blood sugar testing, smoking, foot care, and intake of medications) DES-SF^28^ measures empowerment and attitudes toward diabetes EQ-5D-5L^29^ captures health-related quality of life (including mobility, self-care, usual activities, pain/discomfort, anxiety/mental health) WHO5^30^ capturing the subjective well-being CESD-R^31^ gives hints for depression PAID^32^ discloses problems/dissatisfaction in diabetes treatment
	Patients with either beneficial or nonexistent effect(s), that is, patient groups, who achieve larger or smaller HbA1c changes associated with the use of DHA	HbA1c testing at visit and questionnaires: SDSCA DES-SF
	Reduction in the number of high and low blood sugar readings	Data from DHA or Diary using the following cutoff values/ranges: 80-130 mg/dL (fasting or preprandial)^26^
	Termination rate(s)	Data from DHA and QV1, QV2, QV3 to collect data on patients’ experience and SMBG compliance (one questionnaire for each visit)
H3	Usability for patients in daily self-management	SDSCA UEQ^33^ to capture the user experience
	Number of preprandial and postprandial testing events	Data from DHA or Diary
	Usability and overall satisfaction of patients and HCPs with DHA	CRF (self-made) to assemble all problems with DHA TVQ (self-made) to check the correct handling of SMBG within the telephone call HCP (self-made) shows the evaluation of the physicians’ and also patients’ overall satisfaction with DHA UEQ

Abbreviations: ADA, American Diabetes Association; BG, blood glucose; CESD-R, Center for Epidemilogic Studies Depression Scale - Revised; CG, control group; CRF, case report form; DES-SF, Diabetes Empowerment Scale–Short Form; DHA, digital health application; EQ-5D-5L, Questionnaire of the EuroQol Group in 5-level-version; HCPs, health care professionals; PAID, Problem Areas in Diabetes Scale; QV, Questionnaire for Visit 1/2/3; SDSCA, Summary of Diabetes Self-care Activities Measure; SMBG, self-monitoring of blood glucose; TVQ, Telephone Visit Questionnaire; UEQ, user experience questionnaire; WHO5, Questionnaire of WHO Collaboration Centrein Mental Health.

### Statistical Analyses

An overview of statistical methods is given here, which can be adapted depending on the data situation at the end of the study.

First of all, a summary of baseline data (eg, laboratory and anamnestic data) is essential. Including the 95% confidence interval, categorical variables are presented in absolute and relative frequencies and continuous variables are described by the number of participants, mean, standard deviation, standard error, median, minimum, and maximum.

Parametric and nonparametric tests will be used to compare quantitative variables and frequency, and the chi-square test is applied on qualitative variables. Furthermore the Kolmogorov-Smirnov test will be applied as a test of normal distribution.

We expect the changes in HbA1c value in this study to follow a normal distribution (based on previous other studies).^34^

Analysis of variance (ANOVA) is the main statistical method to analyze the effect of the DHA on HbA1c as primary endpoint to show whether there is a significant difference in primary outcomes between the two treatment groups.^35^ Data from both Visit 2 and Visit 3 will be compared with baseline data from Visit 1. In addition, an ANOVA with repeated measures is performed, and differences between participants as well as the number of visits and a possible interaction between treatment and number of visits are represented by the linear model.

Assuming a significance level of .05, ANOVA can be used to check whether the null hypothesis can be rejected and which alternative hypotheses (Table 2) can be accepted. Possible correlations are determined using the Pearson correlation coefficient.

For multiple imputation of missing data, the jump-to-reference (J2R) method is used, among others. To impute missing single HbA1c values, data from the patient’s file of the treating HCP can be used as a substitute (as well as from DHA of the IG). If no values are available here, the “Last mean carried forward” method can be used.^36^

#### Sample size calculation

The sample size calculation is performed with the program G*Power.^37,38^ It is assumed that a reduction of 0.4 HbA1c % points (reduction from 8.0 to 7.6) and an HbA1c standard deviation from 0.8 in the CG and 1.0 in the IG are present. This assumption is based on the previous results of the analysis of the AOK START (Systematic Trial with Analysis of Results in Telemedicine) Project.^10,39^ This resulted in an effect size of 0.6625 and a power of 0.95. With this assumption, a sample size of 77 participants in each group (total of 154; dropout already included) will be necessary to show a significant effect. A 20% drop-out rate is estimated based on the experience of a previous study.^34^

## Discussion

The hypothesis to be investigated in this study is that the use of a DHA has a greater impact on the treatment of diabetes and improving glycemic control than standard therapy. For patients, this could mean a great relief in everyday life and improved support in managing their disease.

It is theorized that the provided self-management support, increased motivation, and structured monitoring will have a positive impact on HbA1c levels, frequency of hypoglycemia and hyperglycemia, and mental health.

Through interoperable procedures, patients can be optimally supported during the study. Including the HCP in the study is beneficial because patients get to know about the intervention in their usual setting, that is, routine physician consultation. This way, there is no additional burden of the study, which could skew results, and in addition, HCPs also learn how to use the DHA system and integrate it into their daily practice.^5^

With regard to the current study design, it should be noted that an unblinded RCT design risks biasing the results by various factors. First, selection bias could arise because recruitment is unblinded in physicians’ offices. Selection bias is mitigated by randomization, but, for example, patients who are not very compliant could already be rejected by the physician.^40^ To avoid this, HCPs and practice staff are briefed before recruitment and will receive a checklist of inclusion criteria listed by importance.

As the CG is not allowed to use the DHA, these participants also denied its potential benefits. This could lead to an ethical dilemma as a risk of unblinding.^41,42^ Recent studies have uncovered the problem that complete blinding is not possible in studies including a CG to test a digital application.^43^

To compensate for these disadvantages and to minimize the suspected bias, it is part of the closure management to invite the CG to use the DHA after completion of the study.

However, if there is an effect from the intervention on the behavior of the CG, this would underestimate the effect of the DHA rather than overestimate a positive effect.^44^

The usefulness and perceived usability are particularly crucial for the acceptance of the proposed digital application and to improve patient compliance.^45^ On this point, the difficulty of defining “digital literacy” (see inclusion criteria of Table 1) appears. Participants should have a basic knowledge of computers and smartphones, but it is difficult to determine whether subsequent barriers are due to lack of technical knowledge or possible poor manageability. By requiring “digital literacy to use a smartphone,” patients unexperienced in using digital devices (ie, old age)^46^ could hide their possible problems with the app in order not to be excluded from the study or because of social pressure. In addition, it is not possible to control whether patients may receive help from family members at home, which also could distort the results. To avoid this, the test persons have the possibility to receive technical support via telephone contact at any time.

The study makes it possible to collect data on “digital literacy to use a (diabetes) smartphone app” in different age groups via questionnaires CRF (case report form), HCP, and Telephone visit questionnaire (TVQ). This could be obtained as an additional outcome of the study.

Although the DHA is only tested in Germany in this study, an English-language version of the app is already available.

To ensure the best proven product, regulation, standards, and scientific analysis are essential, especially for diseases like diabetes mellitus, which is accompanied by microvasular and macrovasular complications and high mortality rates.^47^ If the DHA is shown to have a greater effect on diabetes therapy than standard care, the product may be classified as “DiGA.”^14^ In Germany, this approval from the BfArM is necessary to allow a physician to prescribe the app as an option of standard diabetes management so that the costs can be borne by the health insurance companies.

Because patients must be able to rely on receiving the best possible treatment,^48^ we will detect and eliminate limitations of the application depending on the results of this study.
